# Calcium scoring: a personalized probability assessment predicts the need for additional or alternative testing to coronary CT angiography

**DOI:** 10.1007/s00330-020-06921-7

**Published:** 2020-05-13

**Authors:** Judit Simon, Lili Száraz, Bálint Szilveszter, Alexisz Panajotu, Ádám Jermendy, Andrea Bartykowszki, Melinda Boussoussou, Borbála Vattay, Zsófia Dóra Drobni, Béla Merkely, Pál Maurovich-Horvat, Márton Kolossváry

**Affiliations:** 1 MTA-SE Cardiovascular Imaging Research Group, Heart and Vascular Center, Semmelweis University, Budapest, Hungary; 2 Medical Imaging Centre, Semmelweis University, Budapest, Hungary

**Keywords:** Coronary artery calcium score, Coronary CT angiography, Downstream testing, Image quality, Cardiovascular risk

## Abstract

**Objective:**

To assess whether anthropometrics, clinical risk factors, and coronary artery calcium score (CACS) can predict the need of further testing after coronary CT angiography (CTA) due to non-diagnostic image quality and/or the presence of significant stenosis.

**Methods:**

Consecutive patients who underwent coronary CTA due to suspected coronary artery disease (CAD) were included in our retrospective analysis. We used multivariate logistic regression and receiver operating characteristics analysis containing anthropometric factors: body mass index, heart rate, and rhythm irregularity (model 1); and parameters used for pre-test likelihood estimation: age, sex, and type of angina (model 2); and also added total calcium score (model 3) to predict downstream testing.

**Results:**

We analyzed 4120 (45.7% female, 57.9 ± 12.1 years) patients. Model 3 significantly outperformed models 1 and 2 (area under the curve, 0.84 [95% CI 0.83–0.86] vs. 0.56 [95% CI 0.54–0.58] and 0.72 [95% CI 0.70–0.74], *p* < 0.001). For patients with sinus rhythm of 50 bpm, in case of non-specific angina, CACS above 435, 756, and 944; in atypical angina CACS above 381, 702, and 890; and in typical angina CACS above 316, 636, and 824 correspond to 50%, 80%, and 90% probability of further testing, respectively. However, higher heart rates and arrhythmias significantly decrease these cutoffs (*p* < 0.001).

**Conclusion:**

CACS significantly increases the ability to identify patients in whom deferral from coronary CTA may be advised as CTA does not lead to a final decision regarding CAD management. Our results provide individualized cutoff values for given probabilities of the need of additional testing, which may facilitate personalized decision-making to perform or defer coronary CTA.

**Key Points:**

*• Anthropometric parameters on their own are insufficient predictors of downstream testing. Adding parameters of the Diamond and Forrester pre-test likelihood test significantly increases the power of prediction.*

*• Total CACS is the most important independent predictor to identify patients in whom coronary CTA may not be recommended as CTA does not lead to a final decision regarding CAD management.*

*• We determined specific CACS cutoff values based on the probability of downstream testing by angina-, arrhythmia-, and heart rate–based groups of patients to help individualize patient management.*

**Electronic supplementary material:**

The online version of this article (10.1007/s00330-020-06921-7) contains supplementary material, which is available to authorized users.

## Introduction

Coronary CT angiography (CTA) is a gatekeeper to rule out significant coronary artery stenosis, due to its high sensitivity and negative predictive value [1–3]. In the 2019 European Society of Cardiology Guideline for the diagnosis and management of chronic coronary syndromes, coronary CTA has a class I recommendation as the initial test for symptomatic patients in whom obstructive coronary artery disease (CAD) cannot be clinically excluded [4]. However, in case of irregular heart rate, significant obesity, or inadequate breath-hold, coronary CTA is not suggested since sufficient image quality (IQ) may not be reached (class III recommendation) [4]. Exact cutoff values for clinical factors potentially prohibiting sufficient evaluation of CAD using coronary CTA are scarce. Furthermore, in case of increasing likelihood of obstructive CAD, coronary CTA may not be the ideal choice due to the high pre-test probability of significant CAD [5–9]. However, there is limited information regarding patient characteristics in whom alternative diagnostic test should be applied instead of coronary CTA because of high probability of significant stenosis or foreseeable insufficient IQ of CTA [4].

Coronary calcium score (CACS) correlates with the presence of obstructive CAD and may also identify extensive calcifications, which may further hamper CTA analysis, due to partial volume effects and blooming artifacts [10–12]. However, there is minimal data on whether calcium score values may predict inadequate IQ of coronary CTA.

Therefore, we aimed to assess whether anthropometrics, parameters of the Diamond and Forrester pre-test likelihood test, and CACS can predict the need of further testing after coronary CTA [13]. Further, we wished to provide CACS cutoff values for given probabilities of downstream testing following coronary CTA.

## Materials and methods

### Study participants

We retrospectively included patients who underwent coronary CTA in our Institution between April of 2016 and September of 2019 and who were reported in a structured reporting platform (Axis, Neumann Medical Ltd.) with all required clinical, anthropometric, and imaging data allowing convenient export and analysis of the data. In order to study only patients with suspected CAD, those with prior invasive or non-invasive testing for CAD were excluded from our analysis. We also excluded those who came for CTA of the left atrium and pulmonary veins before atrial ablation therapy; patients under 18 years; those with congenital or structural heart disease; those who underwent open heart surgery or transcatheter valve implantation, or pacemaker implantation; or those in whom evaluation of the CTA scans was not possible due to technical issues such as extravasation of contrast medium. Clinical characteristics and cardiovascular risk factors were obtained by standardized questionnaires before examination.

### CACS measurement and coronary CTA scan protocol

Coronary CACS and CTA examinations were performed on a 256-slice scanner (Brilliance iCT 256, Philips Healthcare) with prospective ECG-triggered axial acquisition mode. For CACS, we used 120-kV tube voltage with 30–50-mAs tube current, and for coronary CTA 100–120 kV with 200–300-mAs tube current depending on patient anthropometrics. Image acquisition was performed with 128 × 0.625-mm detector collimation, and 270-ms gantry rotation time. For heart rate control, a maximum of 50–100 mg metoprolol was given orally and 5–20 mg intravenously, if necessary. In patients with a heart rate of < 80/min, mid-diastolic triggering was applied with 3–5% padding (73–83% of the R-R interval), and in those with ≥ 80/min, systolic triggering was chosen (35–45% of the R-R interval). Iomeprol contrast material (Iomeron 400, Bracco Imaging Ltd.) was used with 85–95 ml contrast agent at a flow rate of 4.5–5.5 ml/s from antecubital vein access via 18-gauge catheter using a four-phasic protocol [14]. Bolus tracking in the left atrium was used to obtain proper scan timing. 0.8 mg sublingual nitroglycerin was given between the CACS and coronary CTA examinations. Non-contrast data sets were reconstructed with a slice thickness and increment of 2.5 mm, while coronary CTA data sets were reconstructed with 0.8-mm slice thickness and 0.4-mm increment.

CACS was measured by a commercially available semi-automated software (HeartBeat-CS, Philips IntelliSpace Portal, Philips Healthcare). Coronary CTA examinations were evaluated by axial, multiplanar, and curved multiplanar reconstructions using commercially available software (Comprehensive Cardiac Analysis, Philips IntelliSpace Portal, Philips Healthcare). All examinations were evaluated physicians with level 3 or equivalent certification for coronary CTA. Luminal stenosis were classified into 6 groups: (1) normal—no luminal stenosis; (2) minimal—< 25% stenosis; (3) mild—25–49% stenosis; (4) moderate—50–69% stenosis; (5) severe—70–99% stenosis; and (6) occluded and were reported in a segment-based fashion into a structured reporting platform (Axis, Neumann Medical Ltd.) based on the guidelines of the Society of Cardiovascular Computed Tomography [15].

### Outcome definitions

We aimed to study the influence of the various clinical parameters and CACS on the need for additional testing of CAD after coronary CTA. Therefore, we determined *Outcome 1* as the need for further testing following coronary CTA due to inadequate IQ (non-diagnostic IQ of at least one coronary segment) and/or the presence of obstructive CAD (at least 50% luminal stenosis on any coronary on CTA). We did further sub-analyses to determine the cause of downstream testing. We determined *Outcome 2* as non-diagnostic IQ of coronary CTA (in at least one coronary segment); and *Outcome 3* as presence of at least 50% luminal stenosis on coronary CTA as these stenoses may be hemodynamically significant [16].

### Model specifications

For all three outcomes, we built the following models. *Model 1*, clinical factors routinely evaluated during coronary CTA: body mass index (BMI), heart rate at scan, and any heart rhythm irregularity (sinus vs irregular) at scan which may reduce coronary CTA IQ. *Model 2*, parameters of *Model 1* and characteristics of the Diamond and Forrester score used to asses pre-test probability of obstructive CAD: age, sex, and type of angina (typical, atypical, non-specific) [13]. *Model 3*, parameters of *Model 2* including also CACS using categories of 0, 1–10, 11–100, 101–400, 401–1000, and > 1000 [17].

### Statistical analysis

Continuous variables are expressed as mean ± SD while categorical variables are expressed as frequencies and percentages. We performed multivariate logistic regression and receiver operating characteristics (ROC) analyses to examine the influence of the various factors (*Models 1–3*) on downstream testing following coronary CTA (*Outcome 1*) and also sub-analyses for the cause of downstream testing: inadequate IQ (*Outcome 2*) or the presence of at least 50% luminal stenosis on coronary CTA (*Outcome 3*). We calculated the *R*^2^ using the Nagelkerke method for the logistic regression models to enumerate the variation accountable to the investigated parameters. Sensitivity, specificity, positive and negative predictive values, and accuracy were derived from ROC analysis using Youden index. We compared the areas under curves (AUCs) for the abovementioned models using the DeLong’s test [18]. Finally, we conducted a simulation analyses, where we predicted the probability of further downstream testing for subgroups of the significant predictors of *Outcome 1*. For this, CACS was included as a continuous variable to allow reporting of exact CACS cutoff values for specific probabilities. All analyses were done in the R environment (version: 3.6.1) [5]. ROC analyses and *R*^2^ values were calculated using the ‘pROC’ (version: 1.15.3) and ‘rsq’ (1.1) packages respectively [6, 7]. Two-tailed *p* values smaller than 0.05 were considered significant.

## Results

### Patient characteristics

We included 6705 patients who underwent coronary CTA due to suspected CAD. One thousand one hundred fifty-eight patients were excluded due to known prior CAD, 1160 CTA scans due to imaging of the left atrium and pulmonary veins before atrial ablation therapy. Furthermore, 267 patients were excluded due to the presence of congenital or structural heart disease, and pacemaker electrodes, and due to any other indication of cardiac CTA. After exclusion, the final number of analyzed patients was 4120 (45.7% female, 57.9 ± 12.1 years). The mean BMI of the patients was 28.4 ± 7.7 kg/m^2^, and mean heart rate was 59.9 ± 9.1 bmp. Arrhythmia was present in 152 patients (3.7%). Detailed data on the cardiovascular risk factors and coronary CTA scan parameters are in Table 1*.* Altogether, 275 (6.7%) of the coronary CTA scans were non-diagnostic in at least one coronary segment. The main reasons for inadequate IQ were motion artifact (248/275, 90.2%), image noise (51/275, 18.5%), and heavy calcification (49/275, 17.8%). Obstructive (> 50% stenosis) coronary artery stenosis was detected in 1073/4120 (26.0%) of the included patients. In 1236/4120 (30.0%) of patients, downstream testing was suggested either because of non-diagnostic IQ or because of the presence of > 50% of stenosis on other coronary segments.

**Table 1:** Demographic data

Patient characteristics	Overall (*n* = 4120)
BMI (kg/m^2^)	28.4 ± 7.7
Frequency (beats/min)	59.9 ± 9.1
Heart rhythm irregularity, *n* (%)	152 (3.7)
Age (years)	57.9 ± 12.1
Female, *n* (%)	1884 (45.7)
Type of angina, *n* (%)	
Non-specific	1882 (45.7)
Atypical	1818 (44.1)
Typical	420 (10.2)
Total CACS, *n* (%)	
0	1839 (44.6)
1–10	256 (6.2)
11–100	830 (20.1)
101–399	644 (15.6)
400–1000	356 (8.6)
> 1000	195 (4.7)
Hypertension, *n* (%)	2434 (59.1)
Diabetes mellitus, *n* (%)	614 (14.9)
Hyperlipidemia, *n* (%)	1640 (39.8)
Current or former smoker, *n* (%)	1246 (30.2)
Dose length product (mGy × cm)	
For CACS	33.2 ± 15.4
For coronary CTA	285.9 ± 94.7

### Factors contributing to the need for downstream testing following coronary CTA

**Fig. 1:**
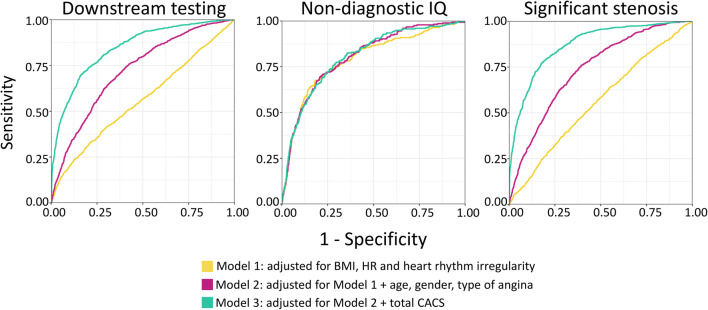
ROC curves of the various models for identifying those who need downstream testing, because of non-diagnostic IQ and/or the presence of significant coronary artery stenosis. Abbreviations: BMI, body mass index; CACS, coronary calcium score; HR, heart rate; IQ, image quality

Anthropometric parameters (*Model 1*) achieved weak diagnostic accuracy to identify patients referred to further testing (AUC = 0.56 [0.54–0.58]). Including parameters of the Diamond and Forrester score (*Model 2*) significantly improved the discriminatory power (AUC = 0.72 [0.70–0.74]; *p* < 0.001). Adding CACS (*Model 3*) further increased the diagnostic accuracy (AUC = 0.84 [0.83–0.86]; *p* < 0.001 compared to *Model 1* and *Model 2*). Sensitivity, specificity, positive and negative predictive values, and accuracy of the models were the following: 39.1%, 72.5%, 73.9%, 73.5%, and 62.5% for *Model 1*; 65.4%, 68.4%, 47.0%, 82.2%, and 67.5% for *Model 2*; and 69.9%, 83.8%, 64.9%, 86.7%, and 79.6% for *Model 3*, respectively. Results are summarized in Fig. 1.

In our final model, each additional beat per minute during the examination increased the odds of downstream testing by 1.03 (*p* < 0.001), the presence of any kind of heart rhythm irregularity by 2.12 (*p* < 0.001), atypical and typical angina by 1.29 and 1.64 (*p* < 0.01; *p* < 0.001; respectively), and CACS categories by CACS_1–10_: 2.46; CACS_11–100_: 4.23; CACS_101–400_: 12.34; CACS_401–1000_: 33.83; and CACS_>1000_: 164.90 (*p* < 0.001 for all). Detailed results can be found in Table 2.

**Table 2:** Multivariate logistic regression models for predicting downstream testing (*Outcome 1*). Abbreviations: *BMI* body mass index; *CACS* coronary calcium score; *OR* odds ratio

Outcome: downstream testing										
		Model 1			Model 2			Model 3		
		OR	95% CI	*p* value	OR	95% CI	*p* value	OR	95% CI	*p* value
BMI		1.01	1.00–1.02	0.02	1.01	1.00–1.02	0.11	0.99	0.98–1.00	0.22
Frequency		1.01	1.00–1.02	< 0.01	1.02	1.01–1.03	< 0.001	1.03	1.02–1.04	< 0.001
Rhythm	Sinus	Reference			Reference			Reference		
	Irregular	2.70	1.93–3.81	< 0.001	1.76	1.22–2.56	< 0.01	2.12	1.39–3.23	< 0.001
Age					1.07	1.06–1.08	< 0.001	1.01	1.00–1.02	0.06
Sex	Female				Reference			Reference		
	Male				2.00	1.72–2.33	< 0.001	1.02	0.85–1.23	0.80
Angina	Non-specific				Reference			Reference		
	Atypical				1.15	0.99–1.34	0.07	1.29	1.08–1.54	< 0.01
	Typical				1.69	1.33–2.15	< 0.001	1.64	1.24–2.17	< 0.001
CACS	0							Reference		
	1–10							2.46	1.70–3.53	< 0.001
	11–100							4.23	3.31–5.42	< 0.001
	101–400							12.34	9.56–15.99	< 0.001
	401–1000							33.83	24.43–47.29	< 0.001
	> 1000							164.90	91.20–325.50	< 0.001
*R*^2^		0.021			0.169			0.423		

### Factors contributing to non-diagnostic image quality resulting in additional testing following coronary CTA

Adding risk factors (*Model 2*) and total CACS (*Model* 3) to anthropometric factors (*Model 1*) did not improve significantly the discriminatory power of finding non-diagnostic IQ of at least one coronary artery segment (AUC = 0.79 [0.78–0.83] for *Model 1*; AUC = 0.80 [0.78–0.83] for *Model 2*; AUC = 0.80 [0.78–0.83] for *Model 3*; all *p* > 0.05). Sensitivity, specificity, positive and negative predictive values, and accuracy of the models for non-diagnostic IQ were the following: 67.3%, 81.9%, 21.0%, 97.2%, and 80.9% for *Model 1*; 69.5%, 79.2%, 19.2%, 97.3%, and 78.6% for *Model 2*; 77.5%, 70.4% 15.8%, 97.8%, and 70.9% for *Model 3*. Results are summarized in Fig. 1.

After adjustment for *Model 3*, each extra beat per minute increased the odds of insufficient IQ by 1.07 (*p* < 0.001), irregular heart rhythm by 2.70 (*p* < 0.001), and CACS groups by CACS_11–100_: 1.53; CACS_101–400_: 2.07; CACS_401–1000_: 2.59; and CACS_>1000_: 3.11 (*p* < 0.05 for all), while male sex decreased the odds of inadequate IQ by 0.54 (*p* < 0.001). Detailed results can be found in *Supplementary Table* *1**.*

### Factors contributing to the presence of significant stenosis resulting in further testing following coronary CTA

Anthropometric parameters (*Model* 1) proved to be insufficient in the discrimination of those with obstructive coronary artery stenosis (AUC = 0.56 [0.54–0.58]). Addition of the age, sex, and type of chest pain (*Model* 2) resulted in significantly higher diagnostic accuracy (AUC = 0.74 [0.72–0.75]; *p* < 0.001). Adding total CACS (*Model* 3) further improved diagnostic power (AUC = 0.87 [0.86–0.88]; *p* < 0.001). For significant coronary artery stenosis, as the outcome, sensitivity, specificity, positive and negative predictive values, and accuracy of the models were the following: 64.1%, 45.0%, 29.1%, 78.1%, and 50.0% for *Model 1*; 75.9% 60.6%, 40.4%, 87.7%, and 64.6% for *Model 2*; and 77.2% 82.3% 60.6%, 91.1%, and 81.0% for *Model 3*.

In the final model, each year of age increased the odds of significant coronary artery lesion by 1.01 (*p* = 0.04), male sex by 1.22 (*p* < 0.01), atypical and typical chest pain by 1.36 and 2.00 (*p* < 0.001), and coronary artery calcification by CACS_1–10_: 4.03; CACS_11–100_: 7.22; CACS_101–400_: 22.16; CACS_401–1000_: 60.34; and CACS_>1000_: 326.75 (*p* < 0.001 for all). Detailed results can be found in *Supplementary Table* *1*.

### Probability of downstream testing for specific CACS thresholds for given heart rates, presence of arrhythmia, and type of chest pain

**Fig. 2:**
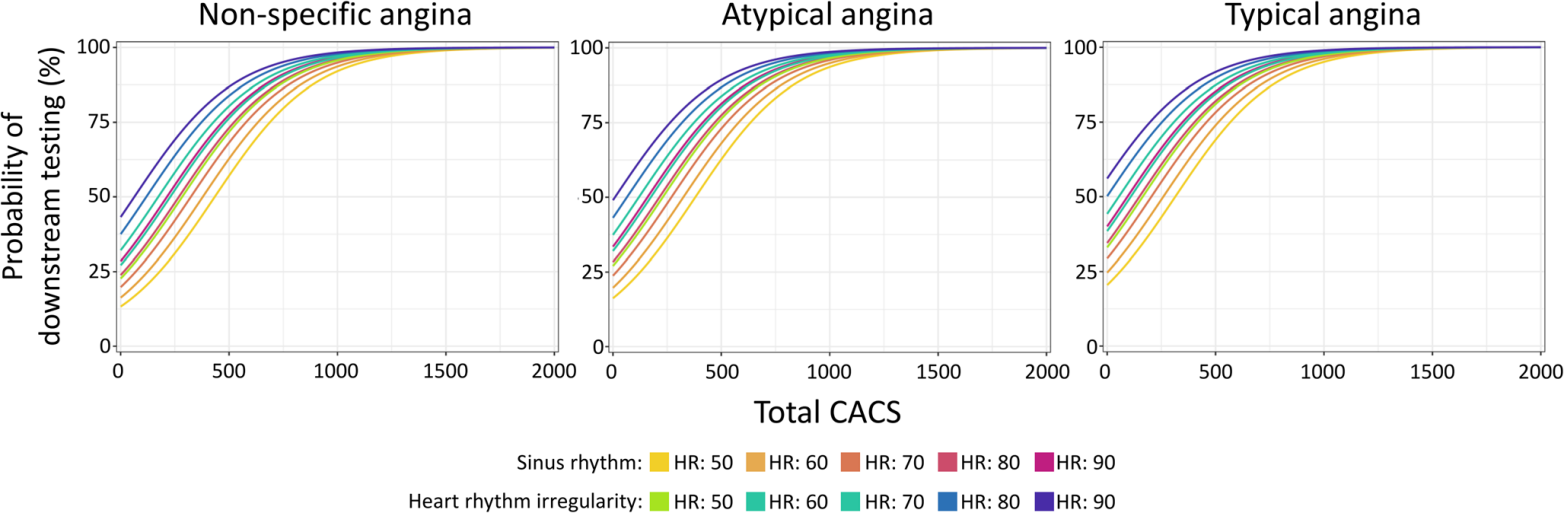
Probability plot of downstream testing following coronary CTA by type of angina, heart rhythm regularity, heart rate, and total CACS. Abbreviations: CACS, coronary calcium score; HR, heart rate

In our analysis of additional testing for any reason after coronary CTA (*Outcome 1*), type of chest pain, heart rate, and presence of heart rhythm irregularity proved to be independent predictors of further testing of CAD beyond total CACS. We simulated the probability of downstream testing for given CACS values for patient groups with non-specific, atypical and typical angina, sinus rhythm, or arrhythmia with heart rate of 50–60–70–80–90 bpm separately. Probability plots are presented in Fig. 2, while CACS cutoff values for specific probabilities of further testing are presented in Table 3*.*

**Table 3:** Specific CACS values for given probabilities for the need of downstream testing by type of angina, heart rhythm regularity and heart rate

		Non-specific angina										Atypical angina										Typical angina									
		Sinus rhythm					Irregular heart rate					Sinus rhythm					Irregular heart rate					Sinus rhythm					Irregular heart rate				
Frequency (bpm)		50	60	70	80	90	50	60	70	80	90	50	60	70	80	90	50	60	70	80	90	50	60	70	80	90	50	60	70	80	90
Probability of downstream testing (%)	50	435	380	325	270	215	285	230	175	166	65	381	326	271	216	161	231	176	121	66	11	316	260	205	150	95	165	110	55	0	0
	80	756	701	646	591	541	606	551	496	441	385	702	647	592	537	482	552	497	442	387	331	636	581	526	471	416	486	431	376	321	266
	90	944	889	834	778	723	794	738	683	628	573	890	835	780	724	669	740	684	629	574	519	824	769	714	659	604	674	619	564	508	453
	95	1117	1062	1006	951	896	967	911	856	801	746	1063	1008	952	897	842	913	857	802	747	692	997	942	887	832	776	847	792	737	681	626

## Discussion

Our results indicate that anthropometric parameters on their own are insufficient to identify patients in whom additional testing of CAD after coronary CTA is highly probable. Classical risk factors of the Diamond and Forrester pre-test probability score gave additional power to the prediction. However, inclusion of total CACS significantly improved the diagnostic accuracy providing excellent discriminatory power to identify patients by whom deferral of coronary CTA may be recommended as a final diagnostic decision regarding CAD could not be made. In case of non-specific angina, CACS above 435, 756, and 944; in atypical angina CACS above 381, 702, and 890; and in typical angina CACS above 316, 636, and 824 correspond to 50%, 80%, and 90% probability of further testing for patients with sinus rhythm of 50 bpm. However, higher heart rates, and presence of heart rhythm irregularity, significantly decrease these cutoffs (*p* < 0.001); therefore, strict heart rate control is still advised. Our results provide individualized cutoff values for given probabilities of downstream testing, which may help personalize decision-making in whom alternative tests may be beneficial as coronary CTA does not lead to a final clinical decision. Future prospective studies are warranted to assess the effect of applying CACS as a gatekeeper for coronary CTA on patient management and outcomes and also on the healthcare system.

One of the new recommendations of the European Society of Cardiology in 2019 is the endorsement of coronary CTA as an initial non-invasive test for those by whom clinical assessment alone is not enough to rule out obstructive CAD [4]. These recommendations follow previous initiatives of the National Institute of Health and Care Excellence in the UK, where coronary CTA is recommended as the initial diagnostic test in stable chest pain patients [9]. In compliance with the guidelines, it is prognosticated that a 700% increase in coronary CTA delivery is required in the UK alone [4]. In order to decrease the burden of the healthcare systems, identification of patients in whom coronary CTA does not lead to a final diagnostic decision-may be needed, as these patients may benefit more from alternative tests.

When analyzing the predictors of further testing of CAD, heart rate, presence of heart rhythm irregularity, type of chest pain, and coronary artery calcium were all significant independent predictors. From all these factors, coronary calcification was by far the most prominent predictor (OR = 33.83 for CACS_401–1000_ and OR = 164.90, for those with CACS_>1000_). These results were mainly driven by the ability of CACS to predict obstructive CAD which needs further testing or intervention, as in our sub-analysis predicting non-diagnostic IQ, the model with simple anthropometric parameters had the same diagnostic accuracy as more complex models incorporating risk factor and CACS.

Coronary artery calcification has been reported to be an important predictor of significant coronary artery stenosis [10, 19–22]. Until now, only some risk-predicting score systems such as the Multi-Ethnic Study of Atherosclerosis risk score implanted CACS into their models, even though a recent study reported that CACS is the most important predictor of CAD-related outcomes [23]. Our results are in line with these findings, since beyond age, male sex, and type of chest pain, CACS proved to be the most prominent predictor of obstructive coronary artery stenosis as depicted by coronary CTA (OR = 60.34 for those with CACS_401–1000_ and OR = 326.75 for CACS_>1000_). These results indicate that routine assessment of CACS before coronary CTA may allow the identification of patients in whom coronary CTA alone may be not enough for the evaluation of CAD, as in these cases the probability of finding at least 50% lumen stenosis potentially being hemodynamically significant and therefore needing further assessment is very high.

Coronary artery calcification may also cause blooming and beam-hardening artifacts resulting in a virtual increase of the plaque volume, and therefore leading to the overestimation of the stenosis and increase of false positive results [12, 24]. Several studies investigated the influence of CACS on the accuracy of coronary CTA. Most of them reported a CACS score > 400 as a threshold for prominent decrease in diagnostic accuracy [7, 25–32]. However, all of them used 64-slice or dual-source CT. Based on our results, in case of a 256-slice CT scanner, CACS did not prove to have additional role in the prediction of insufficient IQ. Above CACS, higher heart rate and arrhythmia were important contributors of insufficient IQ, since in 90.2% of the cases non-diagnostic IQ was due to motion artifact. Moreover, after adjustment for the main cardiovascular risk factors and CACS, heart rate (OR = 1.07), and arrhythmia (OR = 2.70), proved to be important predictors of non-diagnostic IQ. Even though 256-slice CT scanners permit better partial and temporal resolution, rhythm control and optimal heart rate are still important for achieving diagnostic IQ in all coronary artery segments.

Our simulation results revealed that no single CACS cutoff can be provided as the type of chest pain, arrhythmia, and heart rate all significantly change the probability of requiring further downstream testing either because of insufficient IQ or because of presence of significant stenosis. Our tables may help the decision-making at an individual level.

Our study has some limitations. First of all, it is a single-center, retrospective study using only one vendor system; therefore, our results are only generalizable to other populations and other machinery with caution. Second, we have no information on the results of downstream testing. However, our aim was not to assess the diagnostic accuracy of coronary CTA, since the high sensitivity and negative predictive value of this modality are already well-known, but to assess in whom coronary CTA may not be beneficiary as it does not lead to a final diagnostic decision. Third, fractional flow reserve analysis of the CTA images was not done, which could have decreased the referrals to further testing. However, most centers do not have access to this technology yet and therefore fractional flow reserve-based outcomes would limit the generalizability of the results. Finally, it is a single-vendor study; therefore, the effect of anthropometrics might be different on other machines.

In conclusion, routine evaluation of CACS before coronary CTA may be advised as it significantly increases the diagnostic accuracy to identify patients in whom further testing will be needed following coronary CTA. Deferral of these patients to other diagnostic modalities might be beneficial as coronary CTA does not lead to a final decision regarding CAD management.

## Electronic supplementary material

ESM 1 (DOCX 19 kb)

## Abbreviations

AUC: Area under the curve
BMI: Body mass index
CACS: Coronary calcium score
CAD: Coronary artery disease
CI: Confidence interval
CTA: Computed tomography angiography
HR: Heart rate
IQ: Image quality
OR: Odds ratio
ROC: Receiver operating curve
