# Supplementary material for: Calcium scoring: a personalized probability assessment predicts the need for additional or alternative testing to coronary CT angiography
Source: Eur Radiol. 2020 May 13;30(10):5499–506. doi: 10.1007/s00330-020-06921-7 (PMC7476992; doi:10.1007/s00330-020-06921-7)
Supplement: Supplementary file 1 — (DOCX 19 kb) [file 330_2020_6921_MOESM1_ESM.docx]

Supplementary table 1.

|  | | **Outcome: Non-diagnostic image quality** | | | | | | | | | **Outcome: Significant coronary artery stenosis on coronary CTA** | | | | | | | | | |
| --- | --- | --- | --- | --- | --- | --- | --- | --- | --- | --- | --- | --- | --- | --- | --- | --- | --- | --- | --- | --- |
|  |  | **Model 1** | | | **Model 2** | | | **Model 3** | | | **Model 1** | | | | **Model 2** | | | **Model 3** | | |
|  |  | **OR** | **95% CI** | **p value** | **OR** | **95% CI** | **p value** | **OR** | **95% CI** | **p value** | **OR** | **95% CI** | **p value** | | **OR** | **95% CI** | **p value** | **OR** | **95% CI** | **p value** |
| BMI |  | 1.00 | 0.99-1.02 | 0.62 | 1.00 | 0.99-0.02 | 0.59 | 1.00 | 0.98-1.01 | 0.92 | 1.01 | 1.00-1.02 | | <0.01 | 1.01 | 1.00-1.02 | 0.04 | 0.99 | 0.98-1.01 | 0.36 |
| Frequency |  | 1.07 | 1.06-1.08 | <0.001 | 1.07 | 1.06-1.08 | <0.001 | 1.07 | 1.06-1.08 | <0.001 | 0.99 | 0.98-1.00 | | 0.04 | 1.00 | 0.99-1.01 | 0.82 | 1.00 | 0.99-1.01 | 0.51 |
| Rhythm | Sinus | Reference | | | Reference | | | Reference | | | Reference | | | | Reference | | | Reference | | |
|  | Irregular | 3.25 | 2.07-4.99 | <0.001 | 2.64 | 1.66-4.12 | <0.001 | 2.70 | 1.69-4.21 | <0.001 | 1.85 | 1.30-2.62 | | <0.001 | 1.07 | 0.73-1.57 | 0.72 | 1.16 | 0.73-1.81 | 0.06 |
| Age |  |  | | | 1.02 | 1.01-1.04 | <0.001 | 1.01 | 0.99-1.02 | 0.37 |  | | | | 1.08 | 1.07-1.09 | <0.001 | 1.01 | 1.00-1.02 | 0.04 |
| Sex | Female |  |  |  | Reference | | | Reference | | |  |  |  |  | Reference | | | Reference | | |
|  | Male |  |  |  | 0.65 | 0.50-0.86 | <0.01 | 0.54 | 0.40-0.71 | <0.001 |  |  |  |  | 2.48 | 2.11-2.93 | <0.001 | 1.22 | 1.01-1.48 | <0.01 |
| Angina | Non-specific |  |  |  | Reference | | | Reference | | |  |  |  |  | Reference | | | Reference | | |
|  | Atypical |  |  |  | 0.97 | 0.73-1.28 | 0.83 | 0.99 | 0.75-1.31 | 0.94 |  |  |  |  | 1.18 | 1.00-1.38 | <0.05 | 1.36 | 1.12-1.65 | <0.001 |
|  | Typical |  |  |  | 0.92 | 0.58-1.41 | 0.72 | 0.86 | 0.54-1.32 | 0.51 |  |  |  |  | 1.94 | 1.51-2.48 | <0.001 | 2.00 | 1.49-2.70 | <0.001 |
| CACS | 0 |  | | |  | | | Reference | | |  | | | |  | | | Reference | | |
|  | 1-10 |  |  |  |  |  |  | 1.10 | 0.56-2.01 | 0.77 |  |  |  |  |  |  |  | 4.03 | 2.61-6.14 | <0.001 |
|  | 11-100 |  |  |  |  |  |  | 1.53 | 1.02-2.27 | 0.04 |  |  |  |  |  |  |  | 7.22 | 5.34-9.85 | <0.001 |
|  | 101-400 |  |  |  |  |  |  | 2.07 | 1.38-3.10 | <0.001 |  |  |  |  |  |  |  | 22.16 | 16.33-30.39 | <0.001 |
|  | 401-1000 |  |  |  |  |  |  | 2.59 | 1.60-4.14 | <0.001 |  |  |  |  |  |  |  | 60.34 | 42.03-87.78 | <0.001 |
|  | >1,000 |  |  |  |  |  |  | 3.11 | 1.71-5.48 | <0.001 |  |  |  |  |  |  |  | 326.75 | 176.15-658.92 | <0.001 |
| R^2^ | | 0.134 | | | 0.153 | | | 0.167 | | | 0.007 | | | | 0.188 | | | 0.479 | | |
